# Gastroprotective Mechanisms of Action of Semisynthetic Carnosic Acid Derivatives in Human Cells

**DOI:** 10.3390/molecules19010581

**Published:** 2014-01-06

**Authors:** Cristina Theoduloz, Mariano Walter Pertino, Guillermo Schmeda-Hirschmann

**Affiliations:** 1 Laboratorio de Cultivo Celular, Facultad de Ciencias de la Salud, Universidad de Talca, Casilla 747, Talca 3460000, Chile; 2 Laboratorio de Química de Productos Naturales, Instituto de Química de Recursos Naturales, Universidad de Talca, Casilla 747, Talca 3460000, Chile; E-Mails: mwalter@utalca.cl (M.W.P.); schmeda@utalca.cl (G.S.-H.)

**Keywords:** action mechanisms, gastroprotective, carnosic acid, diterpenes

## Abstract

Carnosic acid (CA) and its semisynthetic derivatives display relevant gastroprotective effects on HCl/ethanol induced gastric lesions in mice. However, little is known on the mechanisms of action of the new compounds. The aim of the present work was to assess the gastroprotective action mechanisms of CA and its derivatives using human cell culture models. A human gastric adenocarcinoma cell line (AGS) and lung fibroblasts (MRC-5) were used to reveal the possible mechanisms involved. The ability of the compounds to protect cells against sodium taurocholate (NaT)-induced damage, and to increase the cellular reduced glutathione (GSH) and prostaglandin E_2_ (PGE_2_) content was determined using AGS cells. Stimulation of cell proliferation was studied employing MRC-5 fibroblasts. Carnosic acid and its derivatives **10**–**18** raised GSH levels in AGS cells. While CA did not increase the PGE_2_ content in AGS cells, all derivatives significantly stimulated PGE_2_ synthesis, the best effect being found for the 12-*O*-indolebutyrylmethylcarnosate **13**. A significant increase in MRC-5 fibroblast proliferation was observed for the derivatives **7** and **16**–**18**. The antioxidant effect of the compounds was assessed by the inhibition of lipid peroxidation in human erythrocyte membranes, scavenging of superoxide anion and DPPH discoloration assay. The new CA derivatives showed gastroprotective effects by different mechanisms, including protection against cell damage induced by NaT, increase in GSH content, stimulation of PGE_2_ synthesis and cell proliferation.

## 1. Introduction

Rosemary (*Rosmarinus officinalis* L.) is a shrub occurring in all countries of the Mediterranean basin and introduced into Latin America during the early Spanish conquest. The gastroprotective effect of rosemary extract has been reported [1,2]. Some structure-activity relationships/trends of natural and semisynthetic diterpenes from the plant using the HCl/ethanol induced gastric ulcer lesions in mice have been published [3,4]. Carnosic acid (**CA**) is the main diterpene constituent of rosemary and it also occurs in several Lamiaceae species, including *Salvia officinalis* [5]. Carnosic acid, belonging to the abietane skeleton, has been found to display several biological effects, including gastroprotective [2,4], chemopreventive, anti-inflammatory [6], antioxidant [6,7] and anti-adipogenic activity [8]. Most of the studies on the gastroprotective effect of abietane diterpenes were carried out using either the natural products or their semisynthetic derivatives. Carnosic acid and its semisynthetic derivatives display relevant gastroprotective effect on HCl/ethanol induced gastric lesions in mice [3,4]. At a single oral dose of 10 mg/kg, several of the semisynthetic products presented similar or even better gastroprotective effect than the reference compound lansoprazole at the same dose [4]. However, less is known on the action mechanisms involved in the gastroprotective effect of the products. 

The study of gastroprotective activity of compounds, traditionally carried out using laboratory animals, has incorporated new trends and technologies, reducing the use of laboratory animals. Zheng *et al.* [9] employed the human gastric epithelial cell line AGS to assess the cytoprotective effect of antiulcer compounds. Their results showed a good correlation with previous investigations with primary rat gastric epithelial cells and human studies setting the basis for the use of AGS cell cultures to evaluate antiulcer agents. This cell line consists of mucus-secreting epithelial cells presenting several characteristics of normal gastric epithelial cells, including morphology, microvilli and mucus production. AGS cells, despite being a human gastric adenocarcinoma cell line, still have a good power of differentiation and thus are models used in research related to gastroprotection.

The gastroprotective effect of the studied compounds can be assessed using the bile salts model (sodium taurocholate), considering that bile reflux induces gastric lesions [10,11,12,13]. Free radical generation promotes the appearance of ulcer lesions. This fact explains the ulcerogenic effect of ethanol. An improvement in the antioxidant capacity of gastric cells would render a better protection against oxidative damage and subsequent ulceration [2,14]. Intracellular reduced glutathione (GSH) is an important factor that contributes to the protection of the gastric mucosa against ethanol-induced damage *in vivo* and *in vitro* [15]. In this context, the protective role of GSH in different cultured cells has been reported [13].

Another crucial gastroprotective mechanism involves the prostaglandins (PG) that stimulate multiple defence factors of the gastric mucosa. Prostaglandins accelerate ulcer healing, possibly via angiogenesis, epithelial cell proliferation, production of growth factors, reconstruction of extracellular matrices and suppression of inflammatory cell infiltration [16]. Prostaglandin E_2_ and prostaglandin E_1_ are involved in the synthesis of mucus and bicarbonate, and in the regulation of acid secretion and gastric mucosal blood flow [17].

Furthermore, cell culture models allow the evaluation of selected compounds on the recovery of a pre-existent ulcer lesion. The proliferative capacity of both gastric epithelial cells as well as fibroblasts is a key factor in the renewal and repair of the gastric mucosa, before and after the injury [18]. This process is known as “re-epithelialization”. The aim of this study was to assess the gastroprotective mechanism of **CA** and 18 semisynthetic derivatives using a human gastric adenocarcinoma cell line and lung fibroblast cultures. 

## 2. Results and Discussion

**Figure 1:**
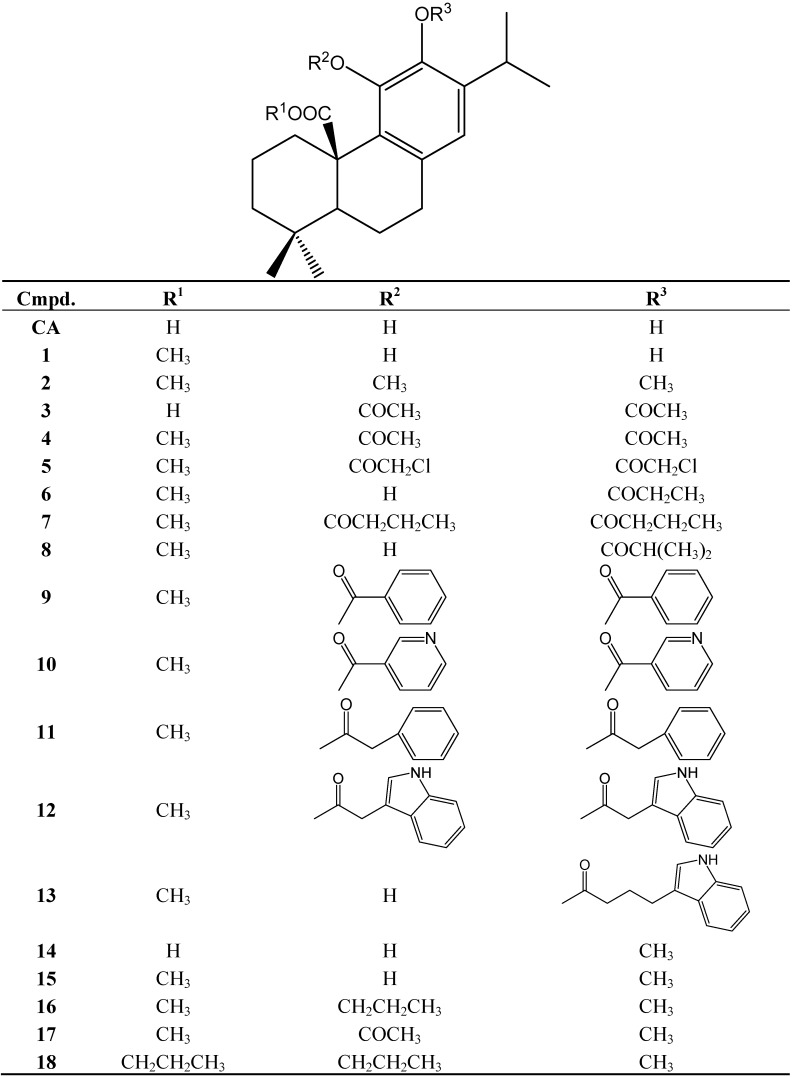
Structure of carnosic acid (CA) and its semisynthetic derivatives **1**–**18**.

In the last years, several reports suggest that the gastroprotective action mechanisms of terpenes are based mainly on the increase in the defensive factors of the gastric mucosa rather than on the inhibition of the gastric aggressive factors (pepsin and HCl secretion) [3,11,19]. Studies carried out with terpenes include the works on the monoterpene limonene and the essential oil of *Citrus aurantium* [20], the diterpene solidagenone [11], as well as the triterpene oleanolic acid [12] and the diterpenes ferruginol [13], carnosic acid [3] as well as the **CA** sources *R. officinalis* [2] and *S. officinalis* [5]. To determine the mechanisms of action of **CA**, several semisynthetic derivatives were prepared and assessed for different possible ways of action using human cell models. The synthesis of the compounds is described in [4]. The structure of **CA** and the semisynthetic derivatives **1**–**18** is presented in Figure 1. The purity of the compounds was >95% as determined by ^1^H-NMR.

### 2.1. Cytotoxicity

The cytotoxicity of **CA** and its derivatives (compounds **1**–**18**) was previously determined and reported in [4]. The cytotoxicity values (IC_50_, µM) were required as a reference to determine the working concentrations in the mechanisms of action experiments.

### 2.2. Sodium Taurocholate-Induced Damage to AGS Cells

**Figure 2:**
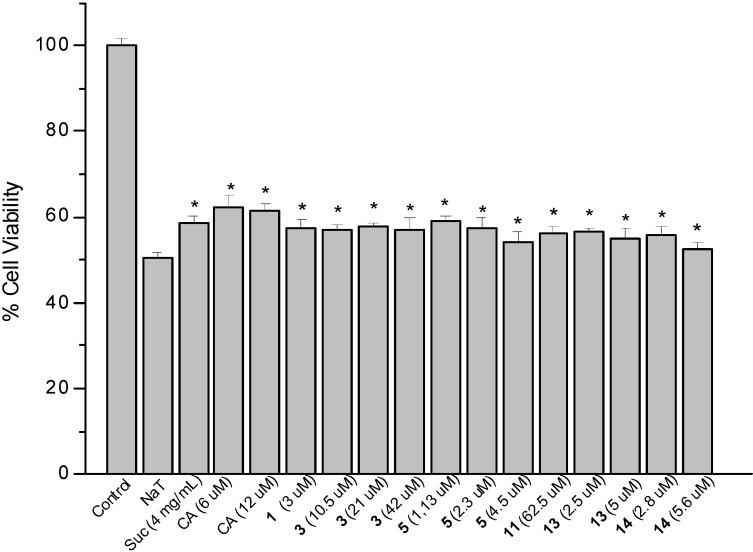
Effect of pre-treatment during 60 min with the reference compound sucralfate (**Suc**), carnosic acid (**CA**) and derivatives **1**, **3**, **5**, **11**, **13** and **14** followed by an incubation during 30 min with 10 mM NaT on the viability of AGS cells determined by the neutral red uptake assay. Each value represents the mean ± SD of three different experiments in quadruplicate. ANOVA followed by Dunnett’s multiple comparison test. * *p* < 0.05 compared to NaT group.

The model of AGS cells damaged by sodium taurocholate (NaT) was used to determine the gastroprotective effect of the compounds against the bile-induced injury on the gastric mucosa [10]. A treatment during 30 min with 10 mM NaT caused a reduction of 50% in cell viability compared to the untreated controls (Figure 2). A pre-treatment during 60 min with **CA** and derivatives **1**, **3**, **5**, **11**, **13** and **14**, at different concentrations, showed a significant cytoprotective effect towards the cell damage caused subsequently by NaT. The percent cytoprotective effect of the compounds, at the effective concentrations and compared to NaT control, was as follows: **CA** (6 and 12 µM, 23 and 22%, respectively), **1** (3 µM, 13%), **3** (10.5, 21 and 42 µM, 13, 15 and 13%, respectively), **5** (1.1, 2.3 and 4.5 µM, 17, 15 and 8%, respectively), **11** (62.5 µM, 12%), **13** (2.5 and 5 µM, 12 and 8%, respectively) and **14** (2.8 and 5.6 µM, 12 and 5%, respectively). Carnosic acid (6 and 12 µM) as well as dichloroacetate **5** (1.1 µM) displayed the same or higher cytoprotective activity than the reference compound sucralfate at 4 mg/mL (580 µM). Pre-treatment with other compounds was not effective. Carnosic acid and the new **CA** derivative **5** presented much better effect than other active terpenes like 12-en-3,11-dioxo-oleanolic acid and 3-β-hydroxysolidagenone and 19-hydroxysolidagenone evaluated using this experimental model [11,12]. Cytoprotective compounds can protect against NaT-induced damage binding bile salts or forming a physical barrier to avoid the mucosal injury [21]. However, other possibilities include interaction with the cell membranes [22] and changes in expression of trefoil factor family 2 mRNA and c-*fos* protein [23].

### 2.3. Determination of Cellular Reduced Glutathione (GSH) Content

Free radical generation promotes the appearance of gastric ulcers, explaining the ulcerogenic effect of ethanol. Intracellular GSH is an important factor contributing to gastric mucosal protection against ethanol-induced damage [15]. A reduction of 50% in the total cellular GSH content was observed in AGS cells treated only with ethanol compared to the controls without ethanol. Carnosic acid and its derivatives **10**–**18** significantly raised GSH levels in AGS cells (Table 1). These compounds comprise esters with heterocycles as well as with aromatic acids and ethers in C-11 and C-12. The most active compounds at the assayed concentrations, expressed as percent increase of GSH content compared with untreated controls, were **CA** (4.5 µM, 19%), the 11,12-*O*-dinicotinylmethylcarnosate **10** (1.4 µM, 14%), 12-*O*-indolebutirylmethylcarnosate **13** (2.0 µM, 11%), 12-*O*-methylcarnosic acid **14** (2.3 µM, 8%) and 12-*O*-metylmethylcarnosate **15** (4.5 µM, 11%). These values are similar to that of the reference compound *N*-acetyl-L-cysteine (**NAC**) at 750 µM (11%), a known stimulant of GSH synthesis. In previous experiments with rats we observed that the diterpene ferruginol counteracted the fall in GSH levels induced by the oral administration of ethanol [24]. 

Esculin (6,7-dihydroxycoumarin-6-*O*-glucoside) is a known hydroxy radical scavenger and inhibitor of liver lipid peroxidation, presenting also anti-inflammatory effect. When evaluated in mice, esculin significantly decreased the lipid membrane peroxidation in the lesioned stomach and the antioxidant effect was also seen in the reduction of the amount of malondialdehyde [25]. The effect of esculin in mice was mediated by endogenous PG synthesis, and release of nitric oxide as well as antioxidant effects [25]. Like the diterpene **CA**, esculin also presents two phenolic hydroxy groups, one of them binding a glucose moiety, leaving a phenol function for the antioxidant effect.

**Table 1:** Total reduced sulfhydryl (GSH) content in post-confluent AGS cells co-incubated with 4% ethanol and **CA** or derivatives **1**–**18** at different concentrations (IC_50_/20) during 12 h.

Compound	Concentration (µM)	GSH (nmol/10^6^ cells)
**Control**	-	3.6 ± 0.2
**NAC *^a^***	750	4.0 ± 0.2 *
**CA**	4.5	4.3 ± 0.2 *
**1**	2.5	3.6 ± 0.1
**2**	4.0	3.7 ± 0.2
**3**	8.5	3.5 ± 0.2
**4**	50.0	3.4 ± 0.1
**5**	0.9	3.4 ± 0.1
**6**	1.2	3.7 ± 0.2
**7**	50.0	3.6 ± 0.2
**8**	2.4	3.7 ± 0.1
**9**	50.0	3.5 ± 0.1
**10**	1.4	4.1 ± 0.2 *
**11**	50.0	4.2 ± 0.2 *
**12**	32.5	4.3 ± 0.3 *
**13**	2.0	4.0 ± 0.2 *
**14**	2.3	3.9 ± 0.3 *
**15**	4.5	4.0 ± 0.3*
**16**	25.0	4.1 ± 0.2 *
**17**	26.0	4.0 ± 0.1 *
**18**	43.5	4.0 ± 0.2 *

Each value represents the mean ± SD of three different experiments in quadruplicate. ANOVA followed by Dunnett’s test. * *p* < 0.05 compared to control group. *^a^* **NAC** (*N*-acetyl-l-cysteine): reference compound.

### 2.4. Determination of Prostaglandin E_2_ (PGE_2_) Content

It has been reported that some terpenes or their derivatives showing gastroprotective effect exert their activity by stimulating PG synthesis *in vivo* and *in vitro* [11,13,26]. The parent diterpene **CA** did not increase the PGE_2_ content at the assayed concentrations on AGS cells (Table 2). However, all derivatives significantly stimulated PGE_2_ synthesis, at one or both concentrations tested, compared with untreated controls. The 12-*O*-indolebutyrylmethylcarnosate **13** at 20 µM presented a strong cytoprotective effect on AGS cells increasing by 2.7 fold the PGE_2_ content compared to controls. Prostaglandins exert a cytoprotective effect preventing gastric mucosal damage induced by necrotizing agents [27]. This fact might explain the gastroprotective effect observed for the **CA** derivatives in the HC/ethanol induced lesions in mice [4]. Regarding the solidagenone derivatives, solidagen-6β-ol significantly induced PGE_2_ synthesis [11]. Oleanolic acid and its derivatives 3-β-acetoxyoleanolic acid, 3-β-acetoxyoleanolic acid methyl ester and 3,12-dioxo-28,13-oleananolide showed a significant stimulation of PGE_2_ synthesis in AGS cells [12]. The effect of ferruginol on gastric lesions both *in vivo* and *in vitro* was related with an increase of PGE_2_ levels in the gastric mucosal cells [13,24].

**Table 2:** Effect of **CA** and derivatives **1**–**18** on the total PGE_2_ content of post-confluent AGS cells treated during 1 h with the compounds at 1/2 and 1/4 of IC_50_.

Compound	Concentration (µM)	PGE_2_ (pg/mL)
**Control**	-	24.9 ± 2.1
**Indomethacin *^a^***	100	8.5 ± 1.3 *
**CA**	45	Bdl *^b^*
	22.5	Bdl *^b^*
**1**	25	8.1 ± 0.8 *
	12.5	10.0 ± 1.2 *
**2**	40	Bdl *^b^*
	20	29.1 ± 2.7 *
**3**	84	29.2 ± 2.9 *
	42	25.1 ± 1.7
**4**	500	35.5 ± 3.2 *
	250	29.2 ± 2.6 *
**5**	9	28.1 ± 2.7 *
	4.5	29.2 ± 2.4 *
**6**	12	30.3 ± 3.7 *
	6	24.1 ± 1.5
**7**	500	42.1 ± 4.4 *
	250	35.5 ± 3.1 *
**8**	24	27.1 ± 2.9 *
	12	15.5 ± 0.9 *
**9**	500	55.1 ± 4.7 *
	250	58.0 ± 4.5 *
**10**	14	24.1 ± 2.3
	7	30.3 ± 2.9 *
**11**	500	29.2 ± 1.8 *
	250	34.2 ± 2.1 *
**12**	325	16.7 ± 0.8 *
	162.5	32.6 ± 3.3 *
**13**	20	68.0 ± 4.1 *
	10	9.3 ± 0.6 *
**14**	23	16.7 ± 1.1 *
	11.5	23.1 ± 2.5
**15**	45	34.2 ± 3.9 *
	22.5	34.2 ± 4.0 *
**16**	246	38.2 ± 2.9 *
	123	Bdl *^b^*
**17**	26	31.5 ± 2.6 *
	13	27.0 ± 2.4 *
**18**	437	34.2 ± 3.3 *
	219	Nd *^c^*

Each value represents the mean ± SD of three different experiments in quadruplicate. ANOVA followed by Dunnett’s test. * *p* < 0.05 compared to control group. *^a^* Reference compound. Bdl *^b^*: Below detection limit. Nd *^c^*: not determined.

Plaunotol is an acyclic diterpene used to treat gastric ulcers in Japan. The gastroprotective mechanisms of action of plaunotol were investigated employing rat gastric epithelial cells (RGM1) [28]. The results showed that plaunotol increased PGE_2_ production and COX-2 expression. In animal models, this diterpene inhibits neutrophil activation preventing indomethacin-induced gastric lesions. Therefore, this mechanism appears to be common in several of the terpenes investigated so far. On the other hand, the activity on PG synthesis was also observed for compounds of very different biosynthetic origin. The inositol derivative quebrachitol (2-*O*-methyl-L-inositol) from *Magonia glabrata* fruits was shown to protect against gastric lesions induced by ethanol and indomethacin by increasing endogenous PG and nitric oxide content as well as activating K^+^_ATP_ channels [29]. This finding is interesting since the increase in PG content and antioxidant activity is also observed in a non-terpenoid compound and points out to cytoprotection mechanisms as a common (or at least) widespread mechanism of action of naturally occurring gastroprotective compounds. Hiruma-Lima *et al.* [30] reported the gastroprotective effect of *Curatella americana* extracts and found that this effect was elicited through endogenous SH groups related to gastric mucus production. In addition, the extract increased PGE_2_ levels in treated rats as well as the participation of endogenous SH compounds in the gastroprotective effect. The possible active constituents of this plant are condensed tannins. Of particular interest is the finding that lipid lowering statins also have other beneficial health effects, including antioxidant and anti-inflammatory activity. The gastroprotective action mechanism of simvastatin was recently investigated [31]. It was shown that the gastroprotective action of simvastatin in rat gastric mucosa was mediated by free radical scavenging, increase in PGE_2_ levels as well as nitric oxide production [31].

### 2.5. Proliferation Assay of MRC-5 Fibroblasts

The proliferative capacity both of gastric epithelial cells as well as fibroblasts is a key factor in the renewal and repair of the gastric mucosa [18]. Tarnawski *et al.* [32] pointed out that ulcer healing is a complex and tightly regulated process of filling the mucosal wound with proliferating and migrating epithelial and connective tissue cells. In order to evaluate the ability of the compounds to accelerate cell proliferation and hence gastric wound healing, their effect on the growth of MRC-5 fibroblasts was determined. A significant stimulation on fibroblast proliferation was observed for derivatives **7**, **16**, **17** and **18**, compared to untreated controls (data not shown). Best effect was exhibited by compound **18**, with 16% (32 µM) and 22% (63 µM) compared to untreated controls, respectively. Compound **16** elicited a 18% growth stimulation at 24 µM and 8% at 12 µM, respectively. The effect observed for compounds **7** and **17** was 8% at 16 µM and 7% at 23 µM, respectively.

The derivatives mentioned are either methyl or propyl esters of **CA** with a methoxy group at C-12 and a short side chain at C-11. The triterpene oleanolic acid (**OA**) was able to stimulate the MRC-5 fibroblasts proliferation, explaining at least in part the effect of this compound both *in vitro* as well as *in vivo* [12]. The **OA** derivative 3β,12β-dihydroxy-28,13-oleananolide was able to stimulate cell growth of fibroblasts at a lower concentration than **OA**. The stimulation of cell proliferation was also one of the gastroprotection mechanisms of solidagenone derivatives [11]. Our data indicate that some derivatives of **CA** might accelerate the repair of ulcer lesions, as reported for ferruginol [13]. Both ferruginol and **CA** display the same skeleton, being **CA** a more oxidized compound. 

### 2.6. Antioxidant Activity

Antioxidant activity of the compounds was assessed by three different methods, namely: inhibition of lipoperoxidation in erythrocyte membranes, superoxide anion scavenging and DPPH discoloration assay. The best antioxidant compound from the series, measured by inhibition of lipid peroxidation in human erythrocyte membranes, was the starting compound **CA** with an IC_50_ value of 2.4 µg/mL (Table 3). Methylation of the COOH group at C-20 markedly reduced the antioxidant effect (compound **1**) compared to **CA**. Protection of the phenolic hydroxy group as acetates with a free COOH function at C-20 (compound **3**) renders an almost inactive product. Therefore, both the free COOH and phenolic OH functions are required for the antioxidant effect. The chloroacetate **5** presented an effect comparable to that of compound **1**. While the compound **6** was weekly active, the derivative **8**, differing in an isobutyl instead of a propyl side chain at C-12, was the most effective derivative of CA found for this assay (IC_50_ 13.6 µg/mL). The activity was similar for phenyl and phenetyl esters, as can be seen comparing the activity of compounds **9** and **11**. None of the studied compounds showed effect neither on the superoxide scavenging anion (at 50 µg/mL) nor on the DPPH discoloration assay (at 100 µg/mL).

**Table 3:** Effect of **CA** and derivatives **1**–**18** on the inhibition of the lipoperoxidation in human erythrocyte membranes. *^a^* Percent effect at 500 µg/mL or IC_50_ values (µg/mL).

Compound	Inhibition of the lipoperoxidation *^a^*
**CA**	IC_50_ 2.4 ± 0.18
**1**	IC_50_ 34.4 ± 4.1
**2**	53
**3**	11
**4**	17
**5**	IC_50_ 27.6 ± 3.0
**6**	42
**7**	34
**8**	IC_50_ 13.6 ± 1.48
**9**	IC_50_ 147.2 ± 16.6
**10**	22
**11**	IC_50_ 140.4 ± 12.6
**12**	38
**13**	49
**14**	IC_50_ 186.3 ± 16.9
**15**	IC_50_ 165.0 ± 14.9
**16**	46
**17**	Nd
**18**	Nd
**Catechin *^b^***	IC_50_ 75.4 ± 6.0

Results are expressed as mean values ± SD of three different experiments in triplicate. *^b^* Reference compound. Nd: not determined due to turbidity.

## 3. Experimental

### 3.1. Compounds

Carnosic acid (**CA**) and 12-*O*-methylcarnosic acid (**14**) were isolated from the aerial parts of *Rosmarinus officinalis* L. (rosemary) cultivated in Curico, Region del Maule, Chile. A voucher herbarium specimen (Pertino 001/2007) has been deposited at the Herbario de la Universidad de Talca. Compounds **1**–**13** and **15**–**18** were synthesized following the methodology described in detail in [4].

### 3.2. MRC-5 Cell Culture

Human lung fibroblasts MRC-5 (ATCC CCL-171) were grown as monolayers in minimum essential Eagle medium (MEM), with Earle’s salts, 2 mM L-glutamine and 2.2 g/L sodium bicarbonate, supplemented with 10% heat-inactivated fetal bovine serum (FBS), 100 IU/mL penicillin and 100 µg/mL streptomycin in a humidified incubator with 5% CO_2_ in air at 37 °C. For the subsequent experiments, cells were plated at a density of 2.5 × 10^4^ cells/mL in 96-well plates. 

### 3.3. AGS Cell Culture

Human epithelial gastric cells AGS (ATCC CRL-1739) were grown as monolayers in Ham F-12 medium containing 1 mM L-glutamine and 1.5 g/L sodium bicarbonate, supplemented with 10% heat-inactivated FBS, 100 IU/mL penicillin and 100 µg/mL streptomycin in a humidified incubator with 5% CO_2_ in air at 37 °C. For the subsequent experiments, cells were plated at a density of 2.5 × 10^4^ cells/mL in 96-well plates.

### 3.4. Cytotoxicity Assay

Basal cytotoxicity assay of **CA** and its derivatives was reported previously [4,33]. These cytotoxicity values (IC_50_, µM) were required as a reference to determine the working concentrations in the experiments described below. Since the compounds evaluated present different cytotoxicity values, it is not possible to assess the effect of the different compounds at the same concentrations in the subsequent experiments. The experimental conditions (working concentrations, incubation time, *etc.*) are set to allow the cells to express the desired effect.

### 3.5. Sodium Taurocholate-induced Damage to AGS Cells

The effect of sodium taurocholate (NaT) on cell viability was determined according to Romano *et al.* [10]. Briefly, one day post-confluent AGS cells were incubated during 60 min with the compounds at 1/4, 1/8 and 1/16 of the respective IC_50_ values. Then, 10 mM NaT was added to all wells for 30 min. Un-treated cells were used as controls. Sucralfate (4 mg/mL) was used as reference compound. After incubation, the neutral red uptake (NRU, 0.05 mg/mL) assay was carried out to determine cell viability [4,33].

### 3.6. Determination of Cellular Reduced Glutathione (GSH) Content

One day after confluence, AGS cells were co-incubated with culture medium containing 4% EtOH and the studied compounds for 12 h. Compounds were tested at 1/20 of the respective IC_50_ values. Untreated cells were used as controls. The GSH synthesis stimulant *N*-acetyl-L-cysteine (750 µM) was used as reference substance. After the incubation time, the GSH content was determined using a colorimetric kit (BioAssays Systems, Hayward, CA, USA). Results are expressed as nanomol of soluble reduced sulfhydryls/10^6^ cells.

### 3.7. Determination of Prostaglandin E_2_ (PGE_2_) Content

One day after confluence, AGS cells were treated for 1 h with the compounds at 1/2 and 1/4 of the respective IC_50_ values. A control without compound was included. Indomethacin (100 μM) was used as standard inhibitor of PG synthesis. After incubation, PGE_2_ content was determined by means of a specific enzyme immunoassay kit (RPN 222, Amersham, Little Chalfont, Buckinghamshire, UK) and values were calculated according to the manufacturer instructions. Results are expressed as pg/mL.

### 3.8. Proliferation Assay of MRC-5 Fibroblasts

One day after seeding, cells were treated with medium supplemented with 10% FBS and the studied compounds at concentrations ranging from 1/64 up to 1/2 of the respective IC_50_ values during 4 days. Untreated cells were used as controls. Cell viability was determined at the end of the incubation by means of the NRU assay. The neutral red concentration was 0.05 mg/mL [4,33].

### 3.9. Inhibition of Lipoperoxidation in Erythrocyte Membranes

The inhibition of lipid peroxidation was determined using human erythrocyte membranes [34]. The products were tested at 500 µg/mL. Catechin served as reference compound (Sigma-Aldrich Co., St. Louis, MO, USA, min 98% by TLC).

### 3.10. Superoxide Anion Scavenging

The superoxide anion scavenging capacity of the studied compounds was evaluated at 50 µg/mL according to [34]. Quercetin was used as reference compound (Sigma-Aldrich Co., min 98% by HPLC).

### 3.11. DPPH Discoloration Assay

The free radical scavenging activity of the products was assessed at 100 µg/mL by the discoloration of a methanolic solution of the 2,2-diphenyl-picrylhydrazyl (DPPH) radical [34]. Catechin was the reference compound.

### 3.12. Statistical Analysis

Results were expressed as mean values ± SD. Experiments with MRC-5 and AGS cells were carried out three times using different cell preparations. Each concentration was tested in quadruplicate. Statistical differences between several treatments and their respective control were determined by one-way analysis of variance (ANOVA) followed by the Dunnett’s multiple comparison test. The level of significance was set at *p* < 0.05. Statistical analyses were carried out using the software SPSS 12.0 for Windows.

## 4. Conclusions

The new **CA** derivatives exert their gastroprotective effects by different mechanisms, including cytoprotection against damage induced by NaT, increase in GSH content, stimulation of PGE_2_ synthesis and cell proliferation. These results might explain the gastroprotective activity of CA and its derivatives observed *in vivo* [3]. Our findings using human cell models are in agreement with the results described by other research groups using animals. The presented methodology allows an insight into the possible gastroprotective mechanisms of action of the compounds avoiding experiments with animals, at least in a first stage. Further studies are required to disclose the potential of the new compounds as gastroprotective agents.
